# Comparative genomic analysis of two emergent human adenovirus type 14 respiratory pathogen isolates in China reveals similar yet divergent genomes

**DOI:** 10.1038/emi.2017.78

**Published:** 2017-11-01

**Authors:** Qiwei Zhang, Shuping Jing, Zetao Cheng, Zhiwu Yu, Shoaleh Dehghan, Amirhossein Shamsaddini, Yuqian Yan, Min Li, Donald Seto

**Affiliations:** 1 Guangzhou Key Laboratory of Drug Research for Emerging Virus Prevention and Treatment, Guangdong Provincial Key Laboratory of Tropical Disease Research, School of Public Health, Southern Medical University, Guangzhou, Guangdong 510515, China; 2 Department of Ophthalmology, Howe Laboratory, Massachusetts Eye and Ear Infirmary, Harvard Medical School, Boston, Massachusetts 02114, USA; 3 Laboratory of Emerging Infectious Diseases and Division of Laboratory Medicine, Zhujiang Hospital, Southern Medical University, Guangzhou, Guangdong 510282, China; 4 Chemistry Department, American University, Washington, D.C. 20016, USA; 5 Bioinformatics and Computational Biology Program, School of Systems Biology, George Mason University, Manassas, Virginia 20110, USA

**Keywords:** adenovirus, China, emergent respiratory pathogen, genome

## Abstract

Human adenovirus type 14 (HAdV-B14p) was originally identified as an acute respiratory disease (ARD) pathogen in The Netherlands in 1955. For approximately fifty years, few sporadic infections were observed. In 2005, HAdV-B14p1, a genomic variant, re-emerged and was associated with several large ARD outbreaks across the U.S. and, subsequently, in Canada, the U.K., Ireland, and China. This strain was associated with an unusually higher fatality rate than previously reported for both this prototype and other HAdV types in general. In China, HAdV-B14 was first observed in 2010, when two unrelated HAdV-B14-associated ARD cases were reported in Southern China (GZ01) and Northern China (BJ430), followed by three subsequent outbreaks. While comparative genomic analysis, including indel analysis, shows that the three China isolates, with whole genome data available, are similar to the de Wit prototype, all are divergent from the U.S. strain (303600; 2007). Although the genomes of strains GZ01 and BJ430 are nearly identical, as per their genome type characterization and percent identities, they are subtly divergent in their genome mutation patterns. These genomes indicate possibly two lineages of HAdV-B14 and independent introductions into China from abroad, or subsequent divergence from one; CHN2012 likely represents a separate sub-lineage. Observations of these simultaneously reported emergent strains in China add to the understanding of the circulation, epidemiology, and evolution of these HAdV pathogens, as well as provide a foundation for developing effective vaccines and public health strategies, including nationwide surveillance in anticipation of larger outbreaks with potentially higher fatality rates associated with HAdV-B14p1.

## INTRODUCTION

Adenoviruses are human pathogens, causing infections and diseases of the respiratory, ocular, gastrointestinal, genitourinary, and metabolic systems, including being linked to obesity.^1^ Interestingly, human adenovirus (HAdV) isolates with highly similar genomes are associated with sporadic and limited infections as well as with larger, wide-spread outbreaks, particularly in environments with close-quartered and/or vulnerable populations.^2, 3, 4, 5, 6, 7, 8, 9, 10, 11, 12, 13, 14^ In terms of public health, although HAdV infections may be highly contagious, they are generally associated with high morbidity rates rather than high mortality rates;^13, 15, 16^ therefore, HAdVs do not command the attention shown to more deadly viral pathogens such as HIV, Ebola virus, and SARS, and MERS coronaviruses. However, in children and immunocompromised individuals, particularly organ transplant recipients, HAdV infections may lead to significant rates of mortality.^13, 17, 18, 19^

To date, applying high-resolution genomic and bioinformatic approaches has yielded in-depth and clearer views of the natural variation of HAdVs.^9, 12, 14, 20, 21, 22, 23, 24, 25, 26, 27, 28, 29, 30, 31, 32, 33, 34, 35, 36, 37^ In particular, the molecular evolution and mechanisms of the genesis of emergent HAdV pathogens are being revealed; for example, recombination appears to be a major driving force^14, 20, 21, 22, 23, 25, 26, 29, 38, 39^ and zoonosis has been revealed as the origin for at least one important HAdV respiratory pathogen.^36, 37, 40^ Computational analysis of the genomes from these emergent HAdV pathogens has led to laboratory-based experiments to explore consequences of specific mutations and their roles in pathogenic properties;^22, 27, 29, 41^ for example, examining the nonsynonymous mutation in the fiber gene that potentially affects host receptor binding, which may contribute to the robustness and altered pathogenic properties of the re-emergent HAdV-14p1.^41^

More than 84 genotypes, including all previously characterized serotypes, have been sequenced, characterized, and classified within seven species A–G (http://hadvwg.gmu.edu/).^21, 24, 28, 29, 30, 31, 42^ Of these, species B, C, and E viruses are associated with respiratory diseases.^17, 43^ Furthermore, species B HAdVs have been divided into subspecies B1 and B2, based on their genome similarities and restriction enzyme digestion patterns,^44^ as well as reports of viruses of these two subspecies showing different tissue tropism.^45^ Members of subspecies B1, i.e., HAdV-B3, -B7, -B16, and -B21, are respiratory pathogens, presumably due to their ability to infect cells of the respiratory tract; HAdV-B50 is also a subspecies B1 virus but has not been associated with a specific, if any, disease.^46^ Subspecies B2 comprises HAdV-B11, -B14, -B34, -B35, and -B55; of these, HAdV-B11, -B34 and -B35 are recognized as renal or urinary tract pathogens.^17^ The remaining two, HAdV-B14 and -B55, are respiratory tract pathogens.^2, 14^ HAdV-B55 is a ‘Trojan Horse’ recombinant with a genome that is essentially that of a respiratory pathogen, HAdV-B14, but with the serologic appearance of a renal pathogen.^6, 7, 8, 9, 10, 14^ HAdV-B14 was originally identified in a military trainee population as an acute respiratory disease (ARD) pathogen in The Netherlands in 1955,^2^ with a second occurrence reported in a civilian setting in England in the same year (1955).^3^ Since then and over the course of approximately fifty years, aside from a few sporadic and limited infections, HAdV-B14 has rarely been reported.

Beginning in 2005, HAdV-B14 re-emerged in the U.S., with multiple outbreaks in both civilian and military settings, as a highly contagious respiratory pathogen that was associated with a 76% hospitalization rate and an 18% fatality rate.^47, 48, 49^ This fatality rate was alarming and unusual for a HAdV epidemic, particularly in presumably otherwise healthy adults. The same genome type, HAdV-B14p1, appeared in Europe with two ARD outbreaks in Ireland (2009) and the U.K. (2011).^5, 11, 50^ These resulted in the highest fatality rates reported for HAdV-B14 outbreaks, at 33% and 23%, respectively.^5, 11, 50^ In 2011, this pathogen re-emerged in Canada and caused one death in the three patients hospitalized with ARD.^49^ In China, there were no HAdV-B14 cases reported until 2010, when HAdV-B14 emerged nearly simultaneously in two geographically distinct locations (Guangzhou and Beijing).^12, 32^ Following these two reports, at least three additional HAdV-B14-related ARD outbreaks in China were recorded: One occurred in the Gansu Province (2011) in which 43 students in an elementary school setting presented with febrile respiratory illnesses,^51^ one occurred in Beijing (2012) in which 30 adults presented with severe symptoms that required hospitalization,^33^ and the third occurred in Liaoning Province (2012) in which 24 students in a middle school presented with febrile respiratory illnesses.^52^ Unlike the U.S. 303600 strain, these five China strains did not have fatalities associated with them.

Given the severity of the symptoms and the numbers of afflicted individuals in China, the earlier outbreaks, with associated higher morbidity and fatality rates in the U.S., Canada, and Europe, and the putative transmission of HAdV-B14 from U.S. to Asia (South Korea) by military trainees (2007),^53^ it is critical to determine the relationships between these China isolates and their global counterparts in order using high resolution genome analysis to understand the epidemiology of this re-emergent pathogen. These data will provide a basis for public health preventive measures, including diagnosis, surveillance, and appropriate protocols for limiting outbreaks and prevention. Specific information of the mutation rates, mutation hotspots, and new variations, particularly at their serologic recognition sites, will play a key role in the development of vaccines against these pathogens. This report presents the bioinformatics analysis of the HAdV-B14p1 Guangzhou strain (GZ01; October 2010), which was the first HAdV-B14 strain isolated in China,^12^ and compares it with the contemporaneous Beijing strain (BJ430; December 2010) as well as a subsequent strain isolated in 2012 (CHN2012; February 2012). Provided is a high-resolution view of highly similar yet intriguingly divergent genomes, linking one of these strains with the globally re-emergent strain isolated in the U.S. in 2005, and suggesting at least two co-circulating lineages of type 14.

## MATERIALS AND METHODS

### Cells, virus stock, and DNA preparation

HAdV-B14p1 GZ01was isolated from a throat swab of a 17-month-old child hospitalized with acute suppurative tonsillitis in Guangzhou, China (October 2010).^12^ Viral DNA was extracted using a Viral DNA Extraction Kit (Omega Bio-Tek Inc Corp; Norcross, GA). The protocol for PCR amplification is described earlier.^54^ All the experimental protocols in this study were approved by the institutional ethics committee of Southern Medical University and were carried out in accordance with the approved guidelines. The informed consent for participation in this study was obtained from the guardian of the under-aged participant. Data records of the sample and sample collection are de-identified and completely anonymous.

### Genome sequencing and annotation

The genome sequence of HAdV-B14p1 strain GZ01 was obtained using the Sanger sequencing method following PCR amplification of targeted overlapping 1–2 kb regions that covered the entire genome, as described earlier.^12^ The sequence data, collected with an ABI 3730 Genetic Analyzer, provided an average genome coverage of 3 to 5 folds, with both strands represented. Gaps and ambiguous sequences were PCR-amplified using different primers and resequenced. DNA sequence fragments were assembled using the SEQMAN software from the Lasergene package (DNAStar; Madison, WI) into a single contig. The genome was annotated with an annotation protocol used for the HAdV-C1 genome analysis,^35^ by first dividing the sequence into contiguous 1 kb non-overlapping segments.

### Sequence analysis

Comparison of sequence differences between and spanning the genomes was performed with Genome Mutation Mapper (GMM; unpublished) software developed by the authors. GMM compares two or more genomes for nucleotide differences, noting SNPs and indels of query genomes relative to a reference; the data were confirmed with DNA Sequencher v5.1 (GeneCodes, Inc.; Ann Arbor, MI). Additionally, pairs of genome sequences were aligned using EMBOSS (http://www.ebi.ac.uk/Tools/emboss/) to show sequence identity, which provided a visualization of the alignment. Nucleic acid and amino acid sequence percent identities were calculated using software which was part of the EMBOSS package. Pair-wise comparisons of genomes were performed with the LAGAN (Limited Area Global Alignment of Nucleotides) program of mVISTA (http://genome.lbl.gov/vista/lagan/submit.shtml).^55^

Phylogenetic analysis of select genes and whole genomes was performed using MEGA 5.1.0 (http://www.megasoftware.net/megamac.php), which accepts FASTA files for sequence alignment and uses a Maximum Composite Likelihood method that generated neighbor-joining and bootstrapped phylogenetic trees with 1000 bootstrap replications; for phylogenetic analysis; all other parameters were set by default.

### Genome type identification

*In silico* restriction maps of available genome sequences from all HAdV-14 strains CHN/GZ01/2010, 303600, CHN/BJ430, CHN2012, and prototype de Wit were generated using the Vector NTI 10.3.0 software (Invitrogen Corp.; San Diego, CA, USA) as described in earlier studies.^56, 57, 58^ These included profiles from restriction enzymes *Bam*HI, *Bcl*I, *Bgl*l, *Bgl*II, *Bst*EII, *Eco*RI, *Hin*dIII, *Hpa*I, *Sal*l, *Sma*I, *Xba*I, and *Xho*I (TaKaRa Corp.; China), all of which were chosen in order to be consistent with the original nomenclature system devised by Li and colleagues.^59^ Genome typing of these strains was determined by comparing its *in silico* RE profiles with other HAdV-14 genome types.^4, 34^

### GenBank accession numbers

The genome sequences used for phylogenic analyses are summarized in Table 1, with additional genome typing details given only for the type 14 strains (‘p’ denotes prototype; if no further designation is noted, the strain is the prototype). Sequences used for these studies included the E1A and hexon genes, either deposited as single gene entries in GenBank or which were extracted from the genome files.

## RESULTS

### Nucleotide sequence analysis of HAdV-B14p1 strain GZ01

**Figure 1:**
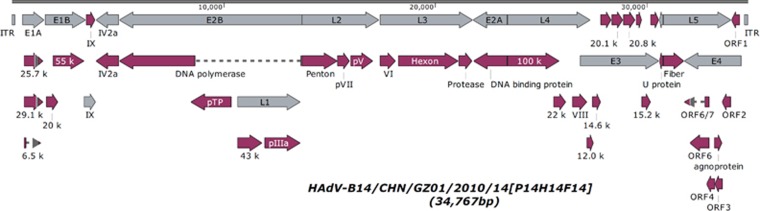
Genomic organization and transcription map of HAdV-B14p1 strain GZ01. The grey arrows indicate the early, intermediate, and late transcription units; the red arrows indicate coding regions. Arrows reflect the direction of the coding transcripts.

The genome of strain GZ01 was sequenced, assembled, annotated, and analyzed using computational methods. Figure 1 presents the genomic organization and transcription map of GZ01. A total of 38 coding sequences were identified. These genome data were deposited in GenBank (accession number JQ824845) under the formal name, preferred by the National Center for Biotechnology Information (NCBI),^24^ ‘Human adenovirus 14 isolate HAdV-B/CHN/GZ01/2010/14[P14H14F14]’ in this report, it is shortened to ‘‘GZ01’’. The genome comprises 34 767 bp, with a GC content of 48.83% that is consistent with the other members of subspecies B2 (mean of 49%).^60^ A contemporaneous isolate BJ430 contains 34 762 bp.^32^ These lengths are very similar to that of other HAdV-B14p1 strains 303600 (Lackland Air Force Base, USA; 2007) and CHN2012 (Beijing, China; 2012),^33^ as well as the prototype from 1955; Genome sizes of 34 763 bp, 34 760 bp, and 34 764 bp, respectively.

### Genome type of strains GZ01, BJ430, and CHN2012 as HAdV-B14p1

**Figure 2:**
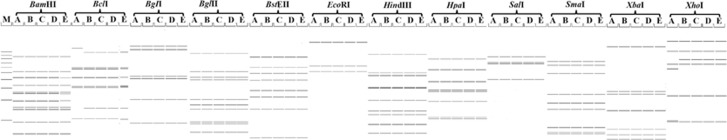
Identification of the genome types of HAdV-B14 strains GZ01 (Guangzhou), BJ430 (Beijing), and CHN2012 (Beijing). The *in silico* restriction enzyme analysis (REA) digestion patterns of HAdV-14 genomes were generated using the Vector NTI 10.3.0 software (Invitrogen Corp., San Diego, CA, USA). Genome digestion patterns of the strains are as follows: (**A**) de Wit or prototype (AY803294); (**B**) GZ01 (JQ824845); (**C**) 303600, reported as HAdV-B14p1 (FJ822614); (**D**) BJ430 (JN032132); and (**E**) CHN2012 (JX892927). These were analyzed with *Bam*HI, *Bcl*I, *Bgl*l, *Bgl*II, *Bst*EII, *Eco*RI, *Hin*dIII, *Hpa*I, *Sal*I, *Sma*I, *Xba*I, and *Xho*I, as described by Li *et al.*^59^ All the three recent China outbreak genomes correspond to the genome type of HAdV-14p1 and have identical REA profiles to each other. They are divergent from the HAdV-B14 prototype (1955). M: 1 kb DNA Sizing Ladder.

Genome type, based on restriction enzyme analysis (REA), was useful in the past for characterizing strains in the absence of full genome sequence data.^59, 61^ It has limited use in the era of whole genome data, but may be useful in comparisons with strains reported in the literature with REA maps but are no longer available for futher analysis. The genome types of strains GZ01, BJ430, and CHN2012 were determined by *in silico* REA using the Vector NTI 10.3.0 software (Invitrogen Corp.; San Diego, CA, USA) and in comparison with the other HAdV-B14 strains ^4, 34^ (Figure 2). The REA profiles of GZ01, BJ430, and CHN2012 were consistent with and indistinguishable from that of HAdV-14p1 strain 303600, all which were confirmed to be genome type 14p1 according to the nomenclature published previously.^4, 34^ It is clear, however, that genome type analysis by REA patterns may be misleading or incomplete as all the three China isolates are highly divergent from HAdV-14p1, as demonstrated by the indel mutations analysis. Furthermore, to support the observation that genome type identification may be misleading, it should be noted that while the initial wet-bench REA data suggested 303600 was a novel HAdV-B14a strain, it was disproven by the whole genome analysis.^4, 34^

### Inverted terminal repeat (ITR)

**Figure 3:**
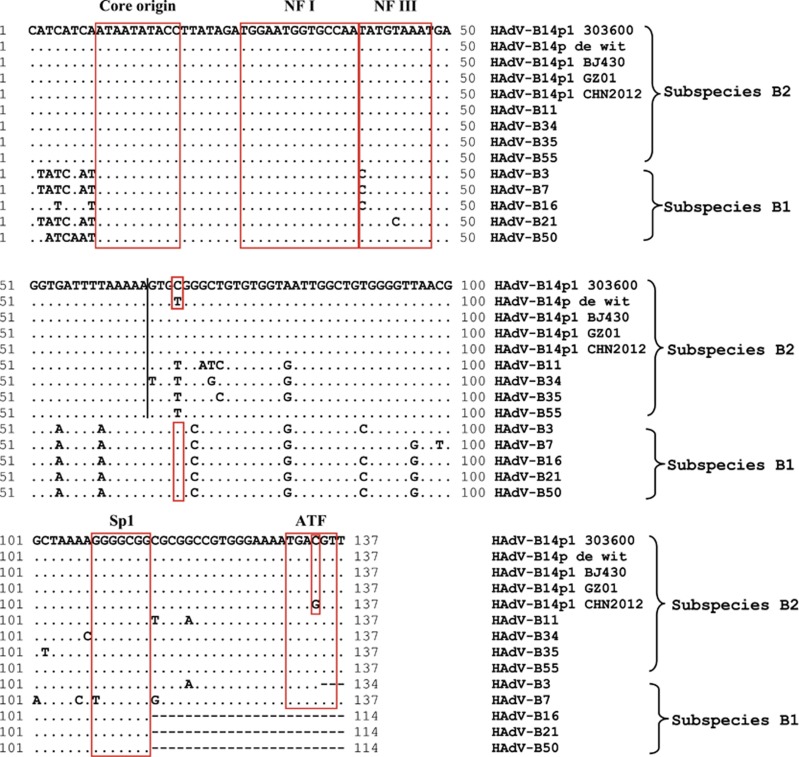
HAdV-B Inverted terminal repeats (ITR) analysis. An alignment of the left ITRs of select HAdV-B prototypes is presented, with two sets of critical sequence motifs (boxed): (1) Replication- Core origin, NF I and NF III binding sites, and (2) Sp1 and ATF binding sites. Nota bene, ‘.’ represent identical bases and ‘-’ represent deletions or gapping for sequence alignment. HAdV-B1: HAdV-B3, -B7, -B16, -B21, and -B50; HAdV-B2: HAdV-B11, -B14, -B34, -B35, and -B55.

ITRs contain sequences encoding critical genome replication functions. The HAdV-B14p1 GZ01 genome has a 137 bp ITR that is identical to the ITRs of the other HAdV-B14p1 strains (BJ430, CHN2012, and 303600), except for a C to G mutation at nt 134 of strain CHN2012 (Figure 3). It is nearly identical to the ITRs reported for the other subspecies B2 members. The first 64 bases are completely identical amongst the prototype subspecies B2 ITRs, which is different for divergent subspecies B1. The binding sites for the host transcription factors NF I and NF III were identified in the ITR.^62, 63^ These sequences have roles in enhancing virus replication and are necessary for efficient virus growth.^62, 63^

Transcriptionally-related Sp1 and ATF binding sites were also conserved in subspecies B2 genomes. All four HAdV-B14p1 genomes differ from HAdV-B14p at position 68, with a C present, a transition mutation from the original T; this is also found at the complementary 3'-end, a validation that it is not a sequencing error. This particular T is conserved across all of the subspecies B2 prototypes, including HAdV-B55, but not amongst all the prototypes of the other subspecies, that is, all subspecies B1 members contain a C at this position; this is a marker to distinguish B1 from B2, and also HAdV-B14p1 from 14p. The nucleotide varies for the other six HAdV species (data not shown).

### Phylogenetic analysis of select genes and the whole genomes

**Figure 4:**
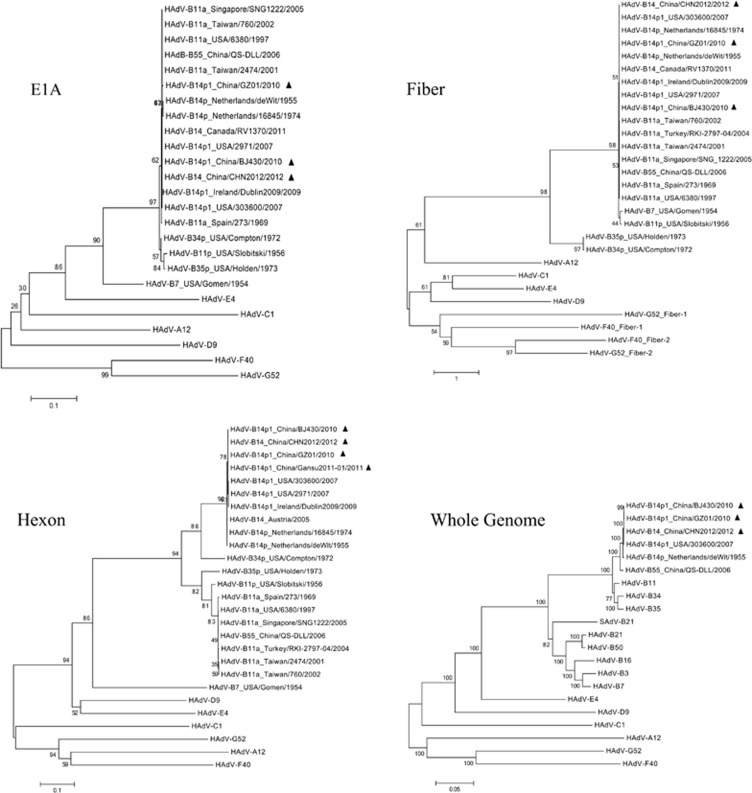
Phylogenetic analysis of the E1A, fiber, and hexon genes, as well as the whole genome sequences, from all HAdV-B14 strains and representatives of other HAdV species. Nucleotide sequences of the E1A, fiber, and hexon genes, as well as limited whole genomes, are available from GenBank. Taxon names include GenBank acc. no., isolation country, strain name, and year of isolation. Phylogenetic trees were generated by using the neighbor-joining method with 1000 replicates and constructed by the MEGA 5.1.0 software (http://www.ebi.ac.uk/tools/mafft). In these analyses, default parameters were applied, with a maximum-composite-likelihood model. Bootstrap numbers shown at the nodes indicate the percentages of 1000 replications producing the clade, with values above 80 considered robust. The scale bar is in units of nucleotide substitutions per site. The China HAdV-B14 strains were indicated with filled triangles.

Of the five recent China strains, only three were reported with whole genome data (GZ01, BJ430, and CHN2012). Phylogenetic analysis of the HAdV-B14 E1A, fiber, and hexon genes shows that the recent HAdV-B14p1 strains (strains GZ01, BJ430, CHN2012, 303600, 2971, and Dublin2009) are closely related to each other as well to the prototype and earlier reported HAdV-B14p genomes (1955 and 1974), as shown in Figure 4. The phylogenetic analysis of the whole genome sequences also confirms the sequence similarity with the prototype genome after approximately 50 years and across large geographical distances (Figure 4).

### Distribution of indel mutations across the genomes

**Figure 5:**
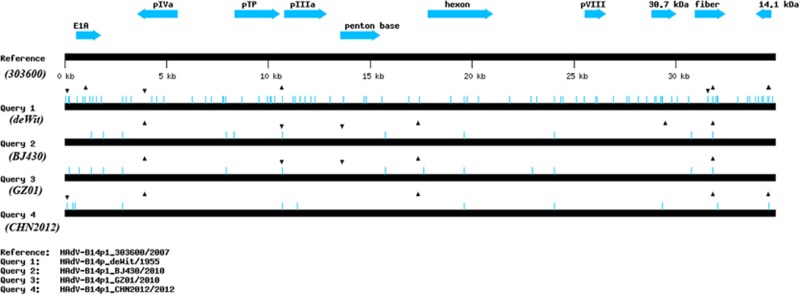
Global genome mutation map visualization of the alignment of genomes from HAdV-14 strains de Wit, 303600, BJ430, GZ01, and CHN2012. To assess the lineages and molecular evolution relationships of these pathogens, Genome Mutation Mapper (GMM; unpublished beta version) is used to provide a global visualization of the nucleotide differences, including insertions and deletions (indels). The contemporaneously circulating and independently isolated HAdV-B14p1 strains have divergent, yet similar mutation profiles, with the indels defining their lineages. For this analysis, the genome of HAdV-14p1_303600/USA/2007 is set as the reference to which the other genomes are compared. Query 1: HAdV-B14p_deWit/The Netherlands/1955 (prototype); Query 2: HAdV-B14p1_BJ430/China//2010; Query 3: HAdV-14p1_GZ01/China/2010; and Query 4: HAdV-B14p1_CHN2012/China/2012. The triangles and inverted triangles indicate deletions and insertions, respectively; blue hash lines indicate base substitutions. Select gene markers are displayed for reference (locations are approximate), as are the genome size markers noted along the bottom.

A global visualization of the alignment of genome sequences of GZ01, BJ430, 303600, CHN2012, and de Wit prototype is presented in Figure 5. This was generated using a ‘beta test version’ software, Genome Mutation Mapper (GMM; unpublished) that notes SNPs and indels of query genomes relative to a reference, and confirmed using DNA Sequencher (GeneCodes, Inc.; Ann Arbor, MI). The sequence similarities amongst them indicates that although these HAdV-B14p1 viruses have a common ancestor (type 14 serotype), their insertion/deletion (indel) patterns suggest two lineages. Indels and their subsequently inherited conserved patterns across genomes are key markers in determining lineages and for following molecular evolution, as reversion is highly unlikely.^64^ Both strains GZ01 and BJ430 are highly divergent from the U.S. strain 303600, with respect to the indel patterns; there is one indel distinguishing between GZ01 and BJ430, a deletion at nt 29 481 in the BJ430 genome, that could be interpreted as GZ01 being more similar to 303600. CHN2012 contains a divergent indel pattern with respect to the other two China isolates. In addition to three deletions in common, it contains two additional indels (one each, insertion and deletion), relative to GZ01 and BJ430, that are uniquely also in the prototype genome. This may be indicative of two sub-lineages and independent introductions into China from abroad, or subsequent divergence from one. The indel numbers and patterns suggest there were at least two prototypes circulating in 1950s, time of the first prototype isolate, with one leading to 303600 and the second leading to the cluster of recent China strains. This visualization of the mutations alignment is critical to the understanding of the molecular evolution and lineages of pathogens as the low resolution genome percent nucleotide identities and REA (Figure 2) present very subtle and limited differences. GZ01, BJ430, CHN2012, and 303600 have highly similar percent identities in comparison with the prototype de Wit at 99.646%, 99.666%, 99.684%, and 99.689%, respectively.

### Base substitution analysis of type 14 strains GZ01, BJ430, CHN2012, 303600, and de Wit

To complement and to check the global genome mutation map of the alignments, each point mutation amongst the five genomes are noted and compared, using the 2007 U.S. strain 303600 as reference (Table 2). Base substitutions are indicative of recent and/or essential mutations as reversion may occur, unlike indel mutations.^64^ The whole genome sequence of strain GZ01 is very similar to that of strain BJ430 with respect to mutations. Six mutation differences are noted between these genomes. These SNPs include two non-synonymous substitutions in the DNA polymerase (K to N) and protein VI genes (R to K). Four synonymous substitutions are noted for the E1A 29.1 K and 25.7 K genes, the L3 VI gene, and the E2A DNA binding protein gene. One mutation is in a non-coding region (nt 225) and two deletions, three T (nt 10 658) and one A (nt 13 266), in poly (T) and poly (A) regions, are also noted (Table 2). Among these mutations, a deletion of A at nt 29 481 in the BJ430 genome distinguishes it from GZ01 (Figure 5).

When compared with strain 303600, 16 base substitutions were found in 11 coding regions of GZ01, which resulted in three non-synonymous substitutions in the E1A 6.5 K, E1B 54.9 K, and Protein VI coding regions, respectively. All of the indels occurred in non-coding regions, which led to length changes of the corresponding poly(A) or poly(T) sequences. Compared with the prototype de Wit strain, there were more mutations in strain GZ01, as would be expected given the time differences between their isolations: 93 base substitutions in the 31 genes, resulting in 35 non-synonymous substitutions. Although there were nine indels involving 25 nucleotides, only two of these resulted in non-synonymous substitutions: One in E1 29.1 K and 25.7 K coding regions (SV to I), with the other resulting the insertion of two amino acids KE in fiber knob domain, which is the most notable sequence difference between the HAdV-14p de Wit and all the other HAdV-14p1 strains (Table 2). This was believed to have the potential of altering cell receptor binding and tissue tropisms, and hence pathogenicity, as the fiber knob recognizes the host cell receptor.^41^

When compared with strain 303600, 11 base substitutions were found in strain CHN2012, which resulted in two non-synonymous substitutions in the E1B 54.9 K, E3 20.8 K coding regions, respectively. There are four synonymous substitutions in the E2B/L1 43 K, hexon, L4 100 K coding regions, respectively. The other mutations located at NCR, including one C to G mutation in ITR.

## DISCUSSION

China’s large, dense, and generally inaccessible population represents a unique environment for studying viral pathogens once they enter the population. The emergence, re-emergence, and transmission of a particular pathogen may be followed using high-resolution genome sequencing and, through computational methods, the molecular evolution and epidemiology of the pathogen may be revealed in great detail. According to the genome analysis of the three HAdV-B14 strains circulating in China, all three strains appear to be of separate, but related, lineages and may likely have been transmitted from abroad. Unlike the past, Beijing and Guangzhou are now ‘open’ to unrestrictive global travel and this may situate them to be the foci for the introduction of infectious disease agents to and from both overseas and other China provinces. Uniquely, Guangzhou has four additional direct long-standing connections to the international community. First, since the mid-1800s, many China emigrants to overseas destinations have originated from this region; therefore, there has ‘always’ been direct physical contact through homecoming visits. Second, Guangzhou has hosted several international events recently, bringing in an influx of visitors. For example, the Asian Games (Nov. 2010) was hosted in Guangzhou, bringing in more than 14 000 athletes along with a large number of foreign tourists and regional transient workers. Third, residents of other China provinces migrate to this prosperous region. Finally, Guangzhou is only 119 km from Hong Kong with the populations commingling; both populations are also in close contact with the global community, including Americans from both hemispheres, neighboring Asians, and Europeans, all of which have experienced respiratory diseases linked to the subspecies B2 HAdVs as well as other respiratory pathogens. For instance, from 2002 to 2003, the severe acute respiratory syndrome coronavirus (SARS-CoV) spread quickly from Guangzhou to Hong Kong and Beijing, and then across the country and globally, with high morbidity and mortality rates. Guangzhou, therefore, serves as an important focal point for infectious disease pathogen introduction, and surveillance is important in order to limit the public health impact on its population and elsewhere in China.

HAdV are important pathogens that appear to re-emerge after lengthy absences, either due to non-reporting because of diminished pathogenicity and infectivity or to perhaps latency or other mechanisms of cryptic infection. HAdV-B14, for example, re-emerged after approximately fifty years and HAdV-B7d re-emerged after twenty-one years.^56^ The 2005 re-emergent HAdV-B14p1 strain was associated with several highly contagious and geographically wide-spread outbreaks of ARD, and included unexpected higher rates of fatalities.^11, 47, 65^ These outbreaks occurred in both civilian (24 communities) and military (nine communities) populations in the United States (2005–2009) and Europe (2009–2011).^4, 5, 11, 47, 50, 65^ Parenthetically and retrospectively, the first case identified was in a child in California (2003)^4^ and was presumably the originating point for the subsequent larger military base outbreaks.^65^

In a limited retrospective survey of respiratory infectious disease agents from patients at a hospital in Guangzhou (2010 and 2011), HAdVs were found and characterized with respect to types.^54^ One of these isolates, typed, deposited, and noted in GenBank as an emergent and previously unreported HAdV type 14 in China (‘human adenovirus 14 isolate HAdV-B/CHN/GZ01/2010/14[P14H14F14’), was isolated from a throat swab of a 17-month-old child with acute suppurative tonsillitis (October 2010). Another simultaneously emergent HAdV-B14 strain BJ430 was isolated from a six-month-old infant diagnosed with bronchial pneumonia and hospitalized at the Beijing Children’s Hospital (December 2010). This is unusual because this condition had not been previously described for infections attributed to either HAdV-B14 or HAdV-B55, with its similar genome and disease symptoms.^11, 14, 48, 66^ As HAdV-B14 has re-emerged globally recently, and since it is known as a highly contagious pathogen that has been associated with high hospitalization rates and fatality rates,^11, 47, 65^ the emergence of this virus should set off an alarm that the proper surveillance of this virus is critical for large dense populations with naive immunity to HAdV-B14.

Comparative genomic analysis is leading to discovery of large numbers of novel molecular markers that are proving very helpful in understanding many important aspects of microbial phylogeny;^67, 68^ of these molecular markers, the conserved indel mutations provide particularly useful means for identifying different groups of microbes in clear molecular terms and for understanding when they have branched off from a common ancestor.^67, 69, 70, 71^ As reported here, indel mutations analysis suggests two co-circulating lineages from at least two co-circulating ‘prototype’ strains in 1950s, one giving rise to the 303600 strain and the other giving rise to the China cluster of HAdV-B14. Before the genomics era, many important adenovirus strains were characterized by REA. REA is still useful for characterizing current isolates by providing a bridge between their accessible genome data and the REA data that are only available in the literature, as important references and as historical isolates are no longer available for genomic analysis.^72^ This report emphasizes that REA data should be carefully considered, as it may be misleading and incorrect. Although the REA analysis confirmed strains GZ01, BJ430, and CHN2012 as belonging to the same genome type 14p1 as strain 303600, high resolution analysis of the genome sequences, including indels, indicate different lineages and introductions into China, along with the absence of fatalities associated with 303600. Again, based on the genome mutation patterns, especially the indels, the de Wit prototype appears to be very different from the 303600 and is similar to the BJ430, GZ01, and CHN2012 strains. These may represent several lineages co-circulating ‘prototype 14’ strains, with one reported in the original study and the rest accumulating characteristic indel patterns that have been reported in this study. Most of the indels occur in the poly(A) and poly(T) regions, which have been proposed as high-resolution molecular strain markers for characterizing HAdV-14p1.^71^ The recent report that HAdV-B14 dispersed from U.S. to Asia in 2007 via military trainees^53^ provides another clue that the emergent HAdV-B14p1 strains in China may have been transmitted from abroad. However, as shown in this report, the whole genome data of that strain is critical for the correct interpretation of that possible transmission pattern.

The whole-genome sequences of strains GZ01 and BJ430 are nearly identical with each other, with respect to point mutations. One indel separates them. Strain GZ01 caused acute suppurative tonsillitis, an upper respiratory disease; however, strain BJ430 was associated with bronchial pneumonia, a lower respiratory tract disease. The reason for this difference in pathogenicity may be due to the genomic differences. A major difference between the two genomes is the two non-synonymous substitutions in the DNA polymerase (K to N) and protein VI genes (R to K), respectively (Table 2). Of these two proteins, the 22-kDa cement protein VI is located beneath the peripentonal hexons in the viral capsid. It is identified as an endosomal membrane lytic factor, which is important for adenoviruses to overcome the barrier of the host cell membrane.^73^ The non-synonymous substitution of R to K in the protein VI may lead to the function of protein VI being enhanced or weakened, which can presumably further change the HAdV tissue tropism. This is awaiting wet-bench studies.

Given the current ease of travel and global interactions, these ARD outbreaks associated with HAdV-B14 provide insights into the distribution, lineage, and molecular evolution of this pathogen. There had been no reports of HAdV-B14 in China prior to and during the period noted for HAdV-B14 re-emergence and circulation (2005–2009). This report presents the bioinformatics analysis of both HAdV-B14p1 strains in high resolution and detail, and provides putative lineages for these two surprisingly and intriguingly *divergent, but, highly similar* genomes linking Guangzhou and Beijing (2294 kilometers apart). A comparison with the re-emergent strain isolated in the U.S. (2007) as well as with the less similar but still remarkably conserved genome of the prototype virus from The Netherlands (1955) is also presented. The presentation of high resolution nucleotide sequence data and a map of mutations, particularly of the indels, provide detailed insight into two contemporaneously circulating human pathogens with divergent and parallel lineages. Both are noted as genome type HAdV-B14p1 via their low resolution but conveniently available REA patterns. The computational data presented in this report again demonstrates highly conserved but divergent HAdV-B14 genomes, which supports further studies of the epidemiology and molecular evolution of these related pathogens, and which, in turn, will provide a foundation for developing effective vaccines and public health strategies, including nationwide surveillance.

## Figures and Tables

**Table 1:** The genome, fiber, hexon, and E1A sequences of adenovirus species A–G used in this study

**Type**	**Strain**	**Year isolated**	**Country**	**Sequence**	**GenBank accession no.**
HAdV-A12	Huie	1954	USA	Genome	AC_000005
HAdV-B3	GB	1953	USA	Genome	AY599834
HAdV-B7	Gomen	1952	USA	Genome	AY594255
HAdV-B11p	Slobitski	1956	USA	Genome	NC_011202
HAdV-B11a	273	1969	Spain	E1A Fiber Hexon	FJ841916 FJ841908 FJ841900
HAdV-B11a	6380	1997	USA	E1A Fiber Hexon	FJ841919 FJ841907 FJ841899
HAdV-B11a	2474	2001	Taiwan	E1A Fiber Hexon	FJ841922 FJ841914 FJ841906
HAdV-B11a	760	2002	Taiwan	E1A Fiber Hexon	FJ841921 FJ841913 FJ841905
HAdV-B11a	RKI-2797-04	2004	Turkey	Fiber Hexon	AY972816 AY972815
HAdV-B11a	SNG1222	2005	Singapore	E1A Fiber Hexon	FJ841920 FJ841912 FJ841904
HAdV-B14p	de Wit	1955	Netherlands	Genome	AY803294
HAdV-B14p	16845	1974	Netherlands	E1A Fiber Hexon	FJ841917 FJ841910 FJ841902
HAdV-B14p1	2971	2007	USA	E1A Fiber Hexon	FJ841915 FJ841909 FJ841901
HAdV-B14p1	Dublin	2009	Ireland	Fiber Hexon	HQ163916 HQ265808
HAdV-B14p1	303600	2007	USA	Genome	FJ822614
HAdV-B14p1	BJ430	2010	China	Genome	JN032132
**HAdV-B14p1**	**GZ01**	**2010**	**China**	**Genome**	**JQ824845**
HAdV-B14p1	CHN2012	2012	China	Genome	JX892927
HAdV-B14	Gansu2011-01	2011	China	Hexon	JX310315
HAdV-B16	ch.79	1955	USA	Genome	AY601636
HAdV-B21	AV-1645	1956	Saudi Arabia	Genome	AY601633
SAdV-21	Bertha	1954	USA	Genome	AC_000010
HAdV-B34p	Compton	1972	USA	Genome	AY737797
HAdV-B35p	Holden	1973	USA	Genome	AY128640
HAdV-B50	Wan	1988	USA	Genome	AY737798
HAdV-B55	QS-DLL	2006	China	Genome	FJ643676
HAdV-C1	Adenoid 71	1953	USA	Genome	AF534906
HAdV-D9	Hicks	1954	USA	Genome	AJ854486
HAdV-E4	RI-67	1952	USA	Genome	AY594253
HAdV-F40	Dugan	1979	Netherlands	Genome	NC_001454
HAdV-G52	T03-2244	2003	USA	Genome	DQ923122

The genome of HAdV-B14p1 GZ01 is shown in boldface.

**Table 2:** Comparison of Mutations Across the Genomes of four HAdV-B14 Strains

**Position**	**Region**	**Product**	**14p deWit**	**14p1 BJ430**	**14p1 GZ01**	**CHN 2012**	**Non-synonymous Substitution**				**Synonymous Substitution**			
							**14p deWit**	**14p1 BJ430**	**14p1 GZ01**	**CHN 2012**	**14p deWit**	**14p1 BJ430**	**14p1 GZ01**	**CHN 2012**
68	ITR	—	C -> T	—	—	—	—	—	—	—	—	—	—	—
134	ITR	—	—	—	—	C -> G	—	—	—	—	—	—	—	—
141	NCR	poly(T)	▾ T	—	—	▾ T	—	—	—	—	—	—	—	—
200	NCR	—	C -> T	—	—	—	—	—	—	—	—	—	—	—
202	NCR	—	C -> T	—	—	—	—	—	—		—	—	—	—
209	NCR	—	G -> C	—	—	—	—	—	—		—	—	—	—
222	NCR	—	T -> C	—	—	—	—	—	—		—	—	—	—
225	NCR	—	—	—	C -> T	—	—	—	—	—	—	—	—	—
403	NCR	—	—	—	—	G -> A	—	—	—	—	—	—	—	—
491	NCR	—	—	—	—	G -> A	—	—	—	—	—	—	—	—
604	E1A	29.1K 25.7K 6.5K	C -> A	—	—	—	L -> M	—	—	—	—	—	—	—
729	E1A	29.1K 25.7K	—	—	G -> A		—	—	—	—			L -> L	
879	E1A	29.1K 25.7K	T -> C	—	—	—	—	—	—	—		—	—	—
887	E1A	29.1K 25.7K	G -> C	—	—	—	G -> A	—	—	—	—	—	—	—
989	E1A	29.1K 25.7K	T -> C	—	—	—	F -> S	—	—	—	—	—	—	—
1028	E1A	29.1K 25.7K	▴GTG	—	—	—	SV -> I	—	—	—	—	—	—	—
1229	E1A	Intron	G -> A	—	—	—	—	—	—	—	—	—	—	—
1299	E1A	29.1K 25.7K	—	A -> G	A -> G	—	—	—	—	—	S -> S	—	S -> S	
1299	E1A	6.5K	—	A -> G	A -> G			H -> R	H -> R		—	—	—	—
1392	E1A	29.1K 25.7K	T -> C	—	—	—	—	—	—	—	L -> L	—	—	—
1537	NCR	—	T -> G	—	—	—	—	—	—	—	—	—	—	—
1538	NCR	—	C -> T	—	—	—	—	—	—	—	—	—	—	—
1553	NCR	—	A -> C	—	—	—	—	—	—	—	—	—	—	—
1801	E1B	20K	C -> T	—	—	—	—	—	—	—	C -> C	—	—	—
1903	E1B	20K	—	T -> C	T -> C	—	—	—	—	—		A -> A	A -> A	
2852	E1B	54.9K	A -> G	A -> G	A -> G	A -> G	K -> R	K -> R	K -> R	K -> R	—	—	—	—
3015	E1B	54.9K	T -> A	—	—	—	—	—	—	—	G -> G	—	—	—
3265	E1B	54.9K	G -> A	—	—	—	A -> T	—	—	—	—	—	—	—
3927	IX	poly(A)	▾AA	▴A	▴A	▴A	—	—	—	—	—	—	—	—
4273	IV2a	IVa2	A -> G	—	—	—	—	—	—	—	L -> L	—	—	—
4515	IV2a	IVa2	A -> G	—	—	—	—	—	—	—	H -> H	—	—	—
4848	IV2a	IVa2	A -> T	—	—	—	H -> Q	—	—	—	—	—	—	—
6248	E2B	Polymerase	C -> T	—	—	—	—	—	—	—	E -> E	—	—	—
6913	E2B	Polymerase	C -> G	—	—	—	E -> Q	—	—	—	—	—	—	—
7207	E2B	Polymerase	G -> C	—	—	—	L -> V	—	—	—	—	—	—	—
7757	E2B	Polymerase	C -> T	—	—	—	—	—	—	—	G -> G	—	—	—
7772	E2B	Polymerase	A -> G	—	—	—	—	—	—	—	V -> V	—	—	—
7877	E2B	Polymerase	C -> T	—	—	—	—	—	—	—	P -> P	—	—	—
7911	E2B	Polymerase		G -> C	G -> C		—	—	—	—	—	S -> S	S -> S	—
8300	E2B	Polymerase	—	C -> G	—	—	—	K -> N	—	—	—	—	—	—
8701	E2B	pTP	A -> G	—	—	—	—	—	—	—	A -> A	—	—	—
9532	E2B	pTP	G -> A	—	—	—	—	—	—	—	I -> I	—	—	—
9943	E2B	pTP	G -> A	—	—	—	—	—	—	—	V -> V	—	—	—
10085	E2B	pTP	G -> T	—	—	—	P -> H	—	—	—	—	—	—	—
10135	E2B	pTP	G -> A	—	—	—	—	—	—	—	I -> I	—	—	—
10303	E2B	pTP	A -> G	—	—	—	—	—	—	—	A -> A	—	—	—
10658	NCR	poly(T)	▾T	▾TT	▾TTTTT	▾T	—	—	—	—	—	—	—	—
10685	E2B/L1	43K	T -> A	T -> A	T -> A	T -> A	—	—	—	—	R -> R	R -> R	R -> R	R -> R
11187	E2B/L1	43K	C -> T	—	—	—	—	—	—	—	T -> T	—	—	—
11304	E2B/L1	43K	T -> C	—	—	—	—	—	—	—	F -> F	—	—	—
11420	E2B/L1	43K	—	—	—	C -> T	—	—	—	—	—	—	—	A -> A
11532	E2B/L1	43K	C -> T	—	—	—	—	—	—	—	R -> R	—	—	—
11805	E2B/L1	43K	A -> G	—	—	—	—	—	—	—	E -> E	—	—	—
12088	E2B/L1	pIII	G -> A	—	—	—	—	—	—	—	V -> V	—	—	—
12268	E2B/L1	pIII	G -> T	—	—	—	—	—	—	—	L -> L	—	—	—
13013	E2B/L1	pIII	T -> C	—	—	—	—	—	—	—	L -> L	—	—	—
13266	E2B/L1	poly(A)	—	▾A	▾AA	—	—	—	—	—	—	—	—	—
13691	NCR	—	C -> T	—	—	—	—	—	—	—	—	—	—	—
14687	L2	PENTON	C -> T	—	—	—	S -> F	—	—	—	—	—	—	—
14788	L2	Penton	A -> G	—	—	—	N -> D	—	—	—	—	—	—	—
15568	L2	VII	T -> C	—	—	—	—	—	—	—	I -> I	—	—	—
15739	L2	VII	—	G -> A	G -> A	—	—	—	—	—	—	L -> L	L -> L	—
16867	L2	pV	T -> C	—	—	—	—	—	—	—	Y -> Y	—	—	—
17348	NCR	poly(A)	—	▴A	▴A	▴A	—	—	—	—	—	—	—	—
17408	L3	VI	G -> A	—	—	—	D -> N	—	—	—	—	—	—	—
17594	L3	VI	—		G -> A		—	—	R -> K	—	—	—	—	—
17618	L3	VI	—	—	G -> A	—	—	—	—	—	—	—	Q -> Q	—
18944	L3	Hexon	G -> A	—	—	—	—	—	—	—	K -> K	—	—	—
19593	L3	Hexon	A -> G	A -> G	A -> G	A -> G	—	—	—	—	K -> K	K -> K	K -> K	K -> K
19748	L3	Hexon	A -> G	—	—	—	I -> M	—	—	—	—	—	—	—
20309	L3	HEXON	A -> G	—	—	—	—	—	—	—	L -> L	—	—	—
21810	NCR	—	T -> C	—	—	—	—	—	—	—	—	—	—	—
22886	E2A	DBP	A -> G	—	—	—	—	—	—	—	G -> G	—	—	—
22923	E2A	DBP	—	—	C -> T	—	—	—	—	—	—	—	K -> K	—
23153	E2A	DBP	G -> A	—	—	—	—	—	—	—	P -> P	—	—	—
24008	L4	100K	T -> C	T -> C	T -> C	T -> C	—	—	—	—	I -> I	I -> I	I -> I	I -> I
24325	L4	100K	C -> T	—	—	—	—	—	—	—	V -> V	—	—	—
24453	L4	100K	G -> A	—	—	—	G -> D	—	—	—	—	—	—	—
25117	L4	100K	A -> G	—	—	—	—	—	—	—	L -> L	—	—	—
25481	L4	100K	T -> C	—	—	—	—	—	—	—	L -> L	—	—	—
26048	L4	22K 33K	G -> A	—	—	—	—	—	—	—	P -> P	—	—	—
26112	L4	22K 33K	G -> A	—	—	—	V -> I	—	—	—	—	—	—	—
26923	L4	VIII	A -> G	—	—	—	—	—	—	—	P -> P	—	—	—
27569	E3	14.6K	G -> A	—	—	—	V -> I	—	—	—	—	—	—	—
27698	E3	14.6K	A -> G	—	—	—	T -> A	—	—	—	—	—	—	—
28128	E3	18.4K	C -> T	—	—	—	—	—	—	—	F -> F	—	—	—
28139	E3	18.4K	A -> C	—	—	—	K -> T	—	—	—	—	—	—	—
28568	E3	20.1K	G -> A	—	—	—	V -> I	—	—	—	—	—	—	—
28952	E3	20.8K	T -> C	—	—	—	V -> A	—	—	—	—	—	—	—
29022	E3	20.8K	G -> A	—	—	—	—	—	—	—	Q -> Q	—	—	—
29223	E3	20.8K	A -> G	—	—	—	—	—	—	—	A -> A	—	—	—
29277	E3	20.8K	A -> T	—	—	—	K -> N	—	—	—	—	—	—	—
29312	E3	20.8K	—	—	—	T -> C	—	—	—	S -> P	—	—	—	—
29335	E3	20.8K	C -> T	—	—	—	P -> S	—	—	—	—	—	—	—
29481	NCR	poly(T)	—	▴T	—	—	—	—	—	—	—	—	—	—
29758	E3	10.1K	T -> C	—	—	—	—	—	—	—	I -> I	—	—	—
30054	E3	15.2K	C -> T	—	—	—	H -> Y	—	—	—	—	—	—	—
30057	E3	15.2K	C -> T	—	—	—	P -> S	—	—	—	—	—	—	—
30621	U	U	G -> A	—	—	—	—	—	—	—	F -> F	—	—	—
30733	U	U	—	G -> A	G -> A	—	—	—	—	—	—	I -> I	I -> I	—
31546	L5	FIBER	▾AGAAAA	—	—	—	▾KE	—	—	—	—	—	—	—
31561	L5	FIBER	C -> T	—	—	—	—	—	—	—	T -> T	—	—	—
31786	NCR	—	—	G-> C	G-> C	—	—	—	—	—	—	—	—	—
31791	NCR	Poly(A)	▴A	▴A	▴A	▴A	—	—	—	—	—	—	—	—
31797	NCR	—	T -> C	—	—	—	—	—	—	—	—	—	—	—
31940	E4	ORF6/7	T -> G	—	—	—	Y -> S	—	—	—	—	—	—	—
31979	E4	ORF6/7	G -> T	—	—	—	A -> D	—	—	—	—	—	—	—
32017	E4	ORF6/7	C -> A	—	—	—	Q -> H	—	—	—	—	—	—	—
32028	E4	—	—	—	—	C -> T	—	—	—	—	—	—	—	—
33005	E4	ORF4	C -> T	—	—	—	R -> K	—	—	—	—	—	—	—
33492	E4	ORF3	G -> T	—	—	—	—	—	—	—	R -> R	—	—	—
33614	E4	ORF2	C -> T	—	—	—	—	—	—	—	L -> L	—	—	—
33874	E4	ORF2	C -> G	—	—	—	E -> Q	—	—	—	—	—	—	—
34016	E4	ORF1	T -> C	—	—	—	—	—	—	—	L -> L	—	—	—
34205	E4	ORF1	G -> C	—	—	—	F -> L	—	—	—	—	—	—	—
34254	E4	ORF1	G -> T	—	—	—	S -> Y	—	—	—	—	—	—	—
34471	NCR	—	G -> A	—	—	—	—	—	—	—	—	—	—	—
34498	NCR	poly(A)	▴A	—	—	▴A	—	—	—	—	—	—	—	—
34534	NCR	—	—	—	—	C -> G	—	—	—	—	—	—	—	—
34549	NCR	—	A -> C	—	—	—	—	—	—	—	—	—	—	—
34553	NCR	poly(A)	▴AA	—	—	—	—	—	—	—	—	—	—	—
34700	ITR	—	G -> A	—	—	—	—	—	—	—	—	—	—	—

HAdV-B14 prototype (de Wit; 1955), GZ01 (2010), BJ430 (2010), and CHN2012 genomes are analyzed with respect to base substitutions (SNPs) and insertions and deletions (indels), using the 2007 U.S. strain 303600 as reference. Nucleotide and amino acid sequence changes are noted, along with their genome locations and coding consequences. Abbreviations and symbols are as follows: inverted terminal repeat, ITR; non-coding region, NCR; insertion, ▾ deletion, ▴ no change or not applicable, -.
